# White Mold: A Global Threat to Crops and Key Strategies for Its Sustainable Management

**DOI:** 10.3390/microorganisms13010004

**Published:** 2024-12-24

**Authors:** Md. Motaher Hossain, Farjana Sultana, Md. Tanbir Rubayet, Sabia Khan, Mahabuba Mostafa, Nusrat Jahan Mishu, Md. Abdullah Al Sabbir, Nabela Akter, Ahmad Kabir, Mohammad Golam Mostofa

**Affiliations:** 1 Department of Plant Pathology, Bangabandhu Sheikh Mujibur Rahman Agricultural University, Gazipur 1706, Bangladesh; hossainmm@bsmrau.edu.bd (M.M.H.); tanbir@bsmrau.edu.bd (M.T.R.); mahabubamimt@gmail.com (M.M.); jahanmishu2612@gmail.com (N.J.M.); shadalsabbir802@gmail.com (M.A.A.S.); nabelaakter98@gmail.com (N.A.); 2 College of Agricultural Sciences, International University of Business Agriculture and Technology, Dhaka 1230, Bangladesh; farjana1s@iubat.edu; 3 Department of Agriculture, Faculty of Science, Noakhali Science and Technology University, Noakhali 3814, Bangladesh; sabiakhan.ag@nstu.edu.bd; 4 Department of Biology, College of Arts, Education & Sciences, University of Louisiana at Monroe, Monroe, LA 71209, USA; 5 Department of Chemistry, State University of New York College of Environmental Science and Forestry, Syracuse, NY 13210, USA

**Keywords:** *Sclerotinia sclerotiorum*, biological control, disease cycle, epidemiology, fungicides, host resistance, molecular diagnosis

## Abstract

White mold, caused by the fungal pathogen *Sclerotinia sclerotiorum* (Lib.) de Bary, is a significant biotic stress impacting horticultural and field crops worldwide. This disease causes plants to wilt and ultimately die, resulting in considerable yield losses. This monocyclic disease progresses through a single infection cycle involving basal infections from myceliogenically germinated sclerotia or aerial infections initiated by ascospores from carpogenically germinated sclerotia. The pathogen has a homothallic mating system with a weak population structure. Relatively cool temperatures and extended wetness are typical conditions for spreading the disease. Each stage of infection triggers a cascade of molecular and physiological events that underpin defense responses against *S. sclerotiorum*. Molecular markers can help rapid diagnosis of this disease in plants. Effective management strategies encompass altering the crop microclimate, applying fungicides, reducing inoculum sources, and developing resistant plant varieties. Integrated approaches combining those strategies often yield the best results. This review discusses the latest insights into the biology, epidemiology, infection mechanisms, and early detection of white mold. This review also aims to provide comprehensive guidelines for sustainable management of this destructive disease while reducing the use of excessive pesticides in crop fields.

## 1. Introduction

White mold caused by *Sclerotinia sclerotiorum* (Lib.) de Bary is among the deadliest sources of plant biotic stress. The disease is widespread and a common problem in temperate, tropical, and dry climates [1]. White mold is also known by various other names, including watery soft rot, cottony rot, cottony soft rot, drop, stem rot, bloom blight, crown rot, and *Sclerotinia* rot [2]. This fungal pathogen has a broad host preference and infects over a few hundred plant species, including some commercially significant crops, flowers, and weeds [2,3,4]. It causes damage to the plant foliage, blossoms, fruits, and roots during the growth period. There have also been reports of crop damage caused by the fungus in cold storage [5] and during transport [6]. While the disease inflicted by *S. sclerotiorum* is typically sporadic, both in terms of time and location, outbreaks are rather common, and losses can be significant. Yield losses as high as 80–100% have been observed in diverse locations, notably temperate climates [7]. The primary causes of plant damage by *S. sclerotiorum* are the nonexistence of functional host resistance, a wide host range, and complications in coping with the disease.

Presently available management strategies frequently fail to provide adequate disease control in commercial crops. *S. sclerotiorum* control in many crops is further complicated by the bi-cyclic type of epidemics, with separate pre- and post-harvest phases evolving in the storage and field [8]. On the other hand, modern epidemiological research has revealed major links between pathogens, plants, and the environment that impact both disease occurrences and control of *S. sclerotiorum*. Monitoring epidemiological factors and early pathogen detection are essential for preventing the spread of disease and enabling effective management practices. DNA- and sensor-based methods have revolutionized plant disease detection, offering reliable detection at the asymptomatic and symptomatic stages [9,10]. Remote sensing tools are extremely helpful in significantly spatializing diagnostic processes [11]. In addition, implementing innovative crop improvement strategies for enhanced host resistance and integrating various methods facilitate optimal control of *S. sclerotiorum*. This review focuses on the recent updates on white mold succession, pathogen biology, potential disease diagnostic markers and sensors, disease epidemiology, and innovative control approaches that have attained satisfactory results in research trials and those extended to commercial use. This review also discusses the use of advanced biotechnological tools like CRISPR/Cas9 to improve host resistance and provide recommendations for integrating different approaches for optimal control of *S. sclerotiorum*.

## 2. Significance and Negative Impacts of White Mold

White mold disease causes widespread detrimental effects on plants. Infection by white mold often leads to a decreased fresh and dry weight of the stem and root [12]. Infected plants also have less chlorophyll than healthy ones. This is because oxalic acid secreted by white mold fungus during infection ruptures chloroplast membranes, leading to chloroplast degeneration. White mold has negative impacts on the vitality and viability of seeds. The detrimental effects of white mold on seed viability and vigor are gradual and inoculum pressure-dependent [12]. In addition, white mold is a significant cause of severe crop yield reductions, with reported losses in the literature ranging from negligible to as high as 100%. Crops such as beans, soybeans, eggplants, lettuce, potatoes, peanuts, and sunflowers are particularly affected, with average annual losses exceeding 1% for these crops [13]. Many of the worst losses occur in intensive cropping areas, where irrigation and proper nutrition generate a lush, dense plant canopy that encourages disease growth. White mold is a soilborne disease that can become a major and persistent problem once established in a field. Yield losses in favorable circumstances frequently exceed 20–35% [7]. However, disease incidences of over 50% and yield losses as high as 80–100% have been observed in various locations, including temperate climates. In grain legumes, white mold can cause yield losses of up to 50% or more, depending on the growing season and other factors [14]. Yield losses by white mold are estimated to be between 147 and 355 kg/ha [15]. It also leads to indirect losses, such as a decline in pod and fodder quality and a decrease in groundnut kernel dry weight and oil content. Reduced yield components (seeds per pod, plants per acre, and 100-seed weight) and diminished seed quality are caused by white mold [16]. The decrease in crop output due to white mold infection is proportional to the intensity of the disease [17]. Management practices employed to contain the disease may incur additional production costs, resulting in significant economic losses. In North Dakota, the United States, the economic impact of white mold between 1991 and 2002 was estimated to be $94 million [18]. However, appropriate measures can be taken to decrease the damage caused by *Sclerotinia* infections to crops.

## 3. Clinical Symptoms and Signs of White Mold

**Figure 1:**
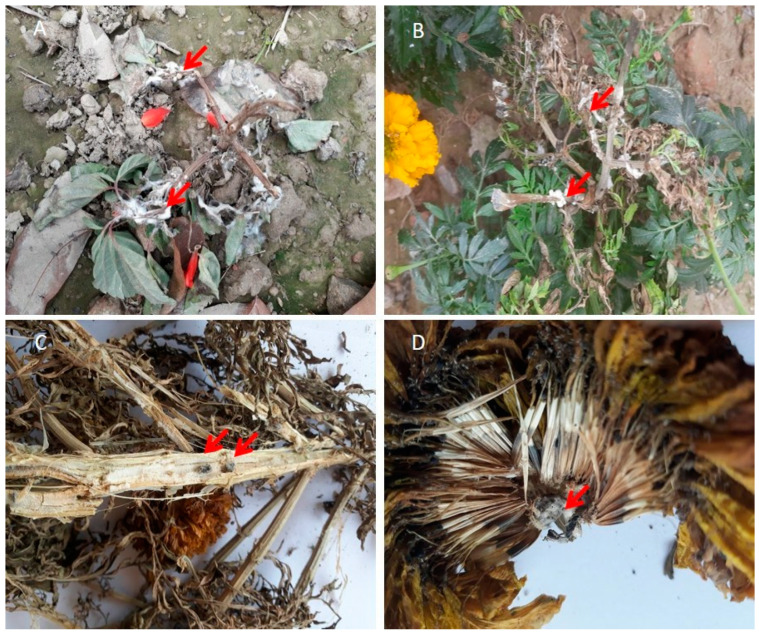
Infected plants showing signs and symptoms of white mold disease. (**A**,**B**) Severely infected salvia (*Salvia splendens*) and marigold (*Tagetes erecta*) plants showing infected, wilted, and dried plant parts with white mycelium growth (arrows indicate). (**C**) Sclerotial development within the pith tissues of an infected stem (arrows indicate). (**D**) An infected marigold flower showing brown discoloration on its outer and inner petals as well as the embedded black sclerotia (arrows indicate).

To effectively manage white mold outbreaks, early detection of signs and symptoms of the disease is essential. Experienced farmers, experts, and agricultural extension workers have traditionally relied on the unique signs and symptoms caused by a pathogen to diagnose the disease. The presence of a white cottony dense mat of mycelial development (a mass of fungus threads) on the host plants and the adjacent soil surface is the most noticeable sign and early symptom of *S. sclerotiorum*, irrespective of plant species (Figure 1; Table 1). The mycelial growth initially appears as a tangled web of fungal threads. Soon, this fluffy white mycelium condenses into large, irregularly shaped white fungal bodies that become dark and brittle as they mature, known as sclerotia. 

Infection typically begins at or near the stem-petiole junction, approximately 10–15 cm above the soil. Early symptoms on infected leaves and petioles appear as dark brown water-soaked patches that quickly spread along the stem and branches. Whitish cottony mycelial masses of the pathogen are seen on the plant foliage, petioles, stems, inflorescence, and pods in the later stages of the disease (Table 1). As the disease advances, infected tissues turn brown and show soft rot, eventually causing the death of the afflicted branches. 

In sunflower, the first aboveground symptom of *S. sclerotiorum* disease is rapid wilting before or during blossoming [19]. Infections on stems can occur at any stage of growth, resulting in stem rot. *S. sclerotiorum* also infects the sunflower head, resulting in the inner head rotting, crumbling, and shredding, leaving huge sclerotia behind [19].

In canola, early flowering followed by wilting at the terminal sections of infected stems is the key symptom of Sclerotinia stem rot (Table 1). Blanched, darkish lesions on the main stems, branches, or pods; and the occurrence of large black sclerotia inside the cortex of infected dead stems are other crucial symptoms and signs [20]. During flowering and seed filling, infected canola stems are frequently prone to lodging. 

White mold also affects many common flowers, including salvia (*Salvia splendens*) (Figure 1A), marigolds (*Tagetes erecta*) (Figure 1B), and cosmos (*Cosmos bipinnatus* and *C. sulphureus*) [2], causing stem rot, wilt, and death. In marigolds and salvia, the first symptoms are usually noticeable during blossoming. Symptoms begin on the flower petals and spread to the entire bloom and the lowest half of the flower (Figure 1C,D). Infection may spread from infected flowers to stems or foliage near the affected blossoms. Infected stems die prematurely, darken, and eventually bleach. The plant completely collapses as the fungus spreads through the stem [21]. Large, dark, irregularly shaped sclerotia are visible on the surface of the host or upon splitting open the dead, infected plant tissues (Figure 1C,D). Under cool and wet conditions, these sclerotia may germinate near the soil surface, producing slender stalks that terminate in small, cup-shaped structures known as apothecia. That is why the formation of apothecia is often seen on the soil surface in the infected field during the foggy winter season.

## 4. Historical Perspective of the White Mold Pathogen

*S. sclerotiorum*, causing white mold, has been recognized for a long time as a significant plant pathogen. However, the taxonomy of this fungus has been a source of confusion from its earliest days, and it has undergone significant taxonomic change and redistribution since its formation by Whetzel [22]. The fungus was initially described as *Peziza sclerotiorum* in 1837 [23], which remained in effect before it was moved to a new genus called *Sclerotinia* [24]. In honor of Libert, the fungus was renamed *Sclerotinia libertania* Fuckel [25]. Both *S. sclerotii* Fuckel and *Peziza sclerotiorum* Lib. were acknowledged as alternative names [26]. *S. libertarian* Fuckel was accepted and utilized by mycologists and plant pathologists until Wakefield [26] demonstrated that it violated the International Code of Botanical Nomenclature, which states that a species moving from one genus to another must keep its former definite name unless the new genus and species combination is by now in use. In this instance, *S. sclerotiorum* remained available. Nevertheless, Wakefield [26] incorrectly stated that G.E. Massee first used the combination *S. sclerotiorum* in 1895. Since then, this organism has been referred to as *S. sclerotiorum* (Lib.) Massee. Later, Purdy [25] pointed out that de Bary first formulated the name in 1884. Hence, *Sclerotinia sclerotiorum* (Lib.) de Bary is the correct name and authority for this fungus.

Aside from the ambiguity about the exact naming, there was also confusion surrounding the type specimen of the fungus [27]. *S. sclerotiorum* (Lib.) de Bary was propositioned for preservation as the type species of the genus by Buchwald and Neergaard in 1976 [28] and recognized as a preserved name in 1981. Whetzel [22] used the criteria of apothecia arising from a definite sclerotium, inoperculate asci usually containing eight ellipsoidal hyaline ascospores, globose spermatia, and lack of conidia to delimit the genus *Sclerotinia*. However, Kohn [28] further delimited *Sclerotinia* to include only those species with the outermost layer of the apothecium (ectal excipulum) constituted of chains of globose cells positioned at right angles to the surface. Based on DNA tests, *S. asari* Wu and Wang and *S. nivalis* Saito were stated as two different species of *Sclerotinia* [29]. However, *S. homoeocarpa* F.T. Bennett was not accepted as valid and had not yet undergone a formal reclassification [30]. *Sclerotinia* is currently the type genus of the Sclerotiniaceae family within the order Helotiales of the phylum Ascomycota. Members of the family are distinguished by the development of brownish, stipitate apothecia with inoperculate asci and unicellular hyaline ascospores originating from a sclerotial stroma on the plant’s outer surface or within plant tissue [31].

## 5. Distinct Morphology and Pathogen Biology

**Figure 2:**
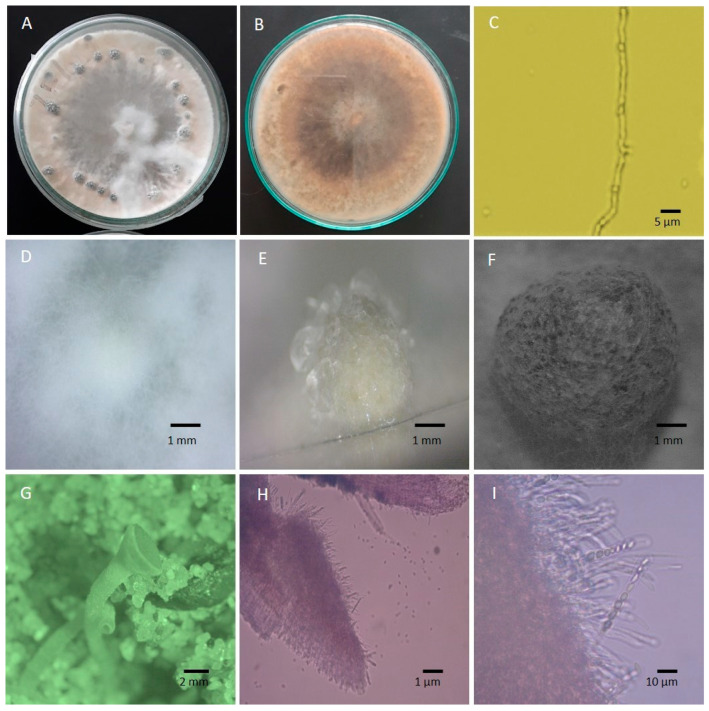
Cultural and morphological features of *Sclerotinia sclerotiorum* causing white mold. (**A**) Pure culture of the infected fungus showing fluffy mycelium and sclerotial ring. (**B**) Salmon buff color is on the opposite side of the colony. (**C**) Micrograph of the mycelium of *S. sclerotiorum* isolated from infected plants. (**D**) Initiation stage of sclerotium (5 days after incubation). (**E**) Development stage of sclerotium (7 days after incubation). (**F**) Maturation stage of sclerotium (10 days after incubation). (**G**) Induced formation of mature apothecia. (**H**) Rows of asci containing ascospores. (**I**) Ascospores in asci.

The white mold fungus *S. sclerotiorum* produces a variety of distinctive morphological and cultural traits, including colony morphology and the characteristics of hyphae, sclerotia, apothecia, asci, and ascospores [32]. Examining these crucial morphological and cultural characteristics of *S. sclerotiorum* is required to identify pathogens accurately. The key cultural and morphological characteristics of *S. sclerotiorum* are often identical across isolates from various hosts, although differences can be observed in colony type, colony color, mycelial diameter, and sclerotial size [33]. Rather et al. [34] studied the cultural characteristics of the 80 isolates from country beans. The colony of different isolates displayed variation in terms of color, pattern, and growth rate. The majority of the isolates gave white colonies, whereas others produced off-white colonies (Figure 2A). The reverse culture on potato dextrose agar (PDA) is salmon buff in color (Figure 2B). Many reports found white to off-white mycelium as an identifier of *S. sclerotiorum* [2,3,35]. However, an *S. sclerotiorum* isolate collected from peanuts in eastern New Mexico is characterized by its dark-colored mycelium on culture media [32]. Only a few isolates of *S. sclerotiorum* have so far been identified with dark-colored mycelium, and they are well known for producing melanin [36]. Dark pigmentation of mycelium is more pronounced when grown on commercially obtained PDA than on a medium prepared from fresh potato broth and agar [32]. It demonstrates that the growth medium significantly influences the pigmentation of *S. sclerotiorum* mycelium. *S. sclerotiorum* colonies on PDA grow quickly and can be fluffy, smooth, or fluffy-at-center-only. However, most isolates produce smooth colonies, while fluffy colonies are primarily produced by isolates from temperate climates [34].

Although mycelium seems white to tan in planta and culture, they are septate, branched, hyaline, and multinucleated under the microscope (Figure 2C) [37,38]. Although *S. sclerotiorum* cannot produce any form of asexual conidia on cultures, it forms large (>2 mm) sclerotia with variable shapes within two weeks. These sclerotia are formed in radiating lines and concentric rings along the growing margins of the colony (Figure 2A). There are three stages of sclerotium formation: commencement or initiation (hyphae amass to create a white sclerotial initial), development (sclerotial initials growth in size), and ripening or maturation (surface delimits, melanin accumulates in peripheral rind cells, and internal cells consolidate) (Figure 2D–F). Mature sclerotium has three layers, making this multi-hyphal structure tough and long-lasting. The thick black outer rind contains melanin, a chemical important in defending against adversarial environments and microbial breakdown [39]. The medulla, the innermost portion, comprises proteins and glucans and is entrenched in a fibrillar matrix. The fungus survives through these multi-hyphal structures (sclerotia) for many years. However, under the right conditions, one or more apothecia, ranging in color from tan to amber, emerge from sclerotium (Figure 2G). A single apothecium bears numerous cylinder-shaped asci that are precisely and closely arranged on the hymenial layer, with each ascus containing eight ascospores (Figure 2H,I). The ascospores are uniseriate, unicellular, hyaline, and elliptical, with numerous paraphyses.

## 6. Monocyclic Disease Cycle

The destructive white mold is a monocyclic disease. *S. sclerotiorum* usually does not produce secondary inoculum and does not spread to other healthy plants during the same season. While *S. sclerotiorum* can move from plant to plant occasionally, this type of transmission is often uncommon. As a versatile organism, *S. sclerotiorum* can operate both as a soil-borne and air-borne pathogen. As a result, *S. sclerotiorum* infections are not rare in the aerial and underground plant tissues. The development of sclerotium is crucial for both cases. A large portion (over 90%) of its life cycle lives as sclerotia [21]. As a result, the most severe disease outbreaks in a field are caused by sclerotia, which remain dormant in the soil for an extended period. The germination of the dormant sclerotium marks the beginning of a new disease cycle. Depending on the intrinsic nature of the fungus and the environmental conditions, the sclerotia either germinate into mycelium (myceliogenic germination) or apothecium (carpogenic germination). In the presence of exogenous nutrients, sclerotia germinate to generate hyphae that invade nonliving organic matter to form mycelium and infect living host tissues. The host cuticle is penetrated through mechanical pressure [40]. Mycelium infects host plants at or below the soil line, but only crowns, roots, and other plant parts within 1 to 2 cm of the sclerotia [13]. Plant parts over 2 cm away from sclerotium are unlikely to be infected by mycelium. Infection of the lateral roots, tap roots, and stem base leads to basal stem rot, wilt, and, in extreme cases, plant death [41].

Carpogenic sclerotia germination leads to the formation of one or more apothecium extending from the sclerotia to the soil surface. During the course of several days, each apothecium is capable of producing millions of ascospores. When the mature apothecia open, hundreds of ascospores are released and readily dispersed by the wind across the plant canopies and the adjoining environment. Ascospores are hyaline and thin-walled and can only subsist a few days after discharge. Ascospores, which are liberated into the air and spread within and between vulnerable agricultural fields, are responsible for the infection of aerial plant plants [42]. Ascospores landing in the extrafloral nectar (sugary secretions) besides the leaf margins germinate and generate hyphae that colonize the leaf, then leafstalk, and, eventually, the stem, causing stem rot in flower plants [41].

**Figure 3:**
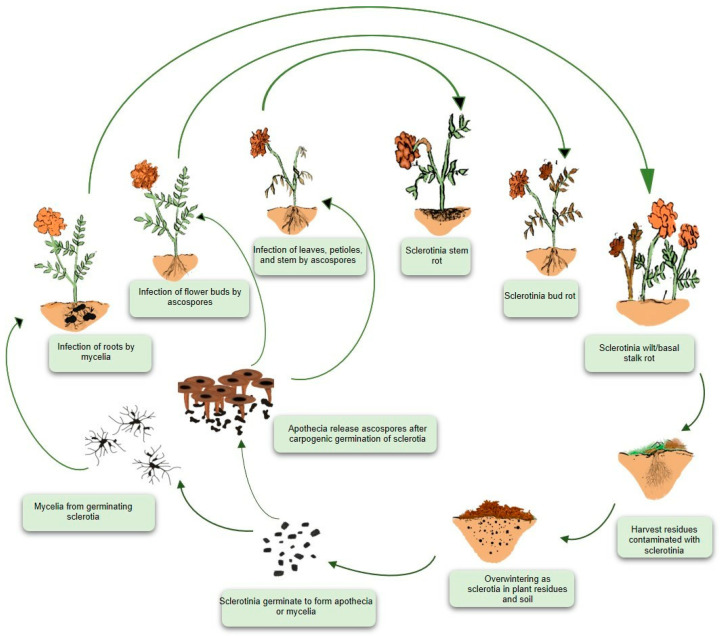
Disease cycle of white mold in marigold. White mold is a monocyclic disease generating only a primary cycle of infection. The causal pathogen *Sclerotinia sclerotiorum* produces a survival structure known as a sclerotium on or inside host tissues, allowing it to thrive in soil. Sclerotia germinate and give rise to apothecia or mycelia. Mycelia infect roots or stem bases, whereas wind-transported ascospores infect aerial parts such as stems, foliage, buds, flowers, and fruits after being liberated from the asci. At the end of the growing season, *S. sclerotiorum* produces sclerotia, which persists on the ground surface or in the soil, on either living or dead plant tissues, until the following season.

During flowering, ascospore deposition in the floral parts can result in capitulum colonization, which causes bud rot (Figure 3). However, ascospores lack the energy to infect healthy tissues directly. Hence, the only way ascospores can germinate, penetrate, and generate compatible infections is to first colonize saprotrophically on dead or senescent tissues and use them as a food base to produce mycelium with sufficient energy to penetrate healthy parts [13]. In field conditions, this process is often accomplished by colonizing senesced, injured, or abscised leaves and flower petals still in physical touch with uninfected intact plant tissues. The fungus completes its life cycle by developing sclerotia in plant cavities such as hollow stems, buds, flowers, and fruits (Figure 3). Once the season is over, the sclerotia remain on the earth’s surface or in the soil, attached to either living or dead plant matter, until next year. These sclerotia enable *Sclerotinia* species to survive harsh environments for extended periods. The melanized black rind acts as a protective barrier, shielding them from microbial invasion and environmental weathering.

## 7. Epidemiological Conditions of the Disease

**Figure 4:**
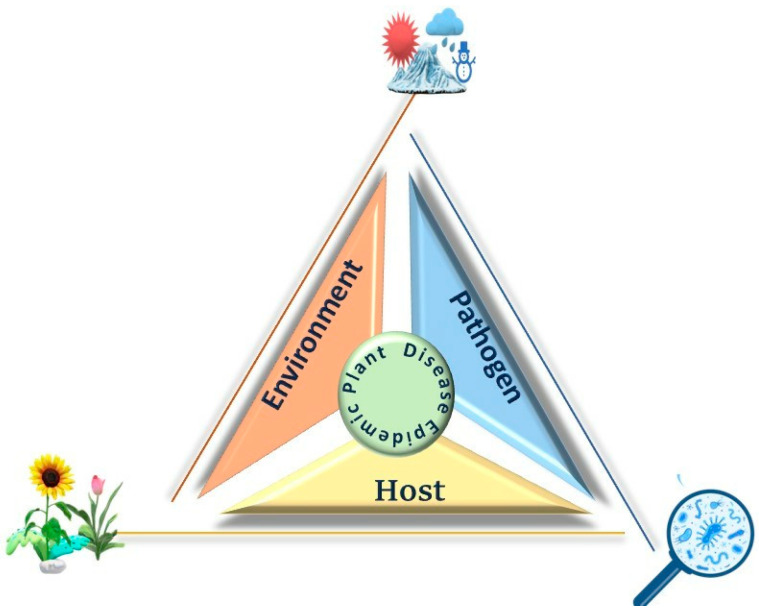
The epidemiological triangle of plant disease showing the interplay between the environment, host, and pathogen. These three factors are interconnected, with the disease outcome depending on their interaction. Environmental conditions, such as temperature and moisture, affect pathogen survival and host susceptibility, leading to the development of plant disease.

The traditional paradigm of the “epidemiological triad” necessitates the interaction of three factors: a susceptible host, a virulent pathogen, and a conducive environment for disease development (Figure 4). The equilibrium of these interactions determines whether or not disease reaches destructive levels in a given situation. Epidemics can only occur when a highly virulent pathogen comes into contact with a susceptible plant host in an environment that favors disease. Understanding these factors and their interactions in a specific locality enables the prediction of disease outbreaks and the implementation of measures to reduce disease prevalence. 

Environmental factors affect both the growth and survival of *S. sclerotiorum* as well as the susceptibility of the host plants. Cool temperatures and moist conditions have been linked to developing white mold disease in various plants [43]. Even though *S. sclerotiorum* can infect plants at any stage of their development, infection most frequently occurs when the plants are in their flowering stages [20]. This is because the optimal conditions for the fungus to develop its apothecia and release its ascospores coincide with those for a fully grown crop canopy, resulting in a cool, moist, and shaded environment for the soil. Under these conditions, the ascospores are able to germinate and infect host tissues successfully [44]. Moreover, flower petals or senescing leaves are particularly vulnerable host tissues and are usually the first to become infected. Therefore, large infection occurrences often coincide with bloom and post-bloom periods, as aging petals fall and lodge in branch axils or attach to leaves, petioles, stems, and developing fruits. In the majority of cases, the conditions necessary for disease development include extended periods of leaf wetness and sufficient canopy moisture. Fog, mist, and dew can provide ample moisture, creating wet and humid weather. 

High planting densities, close row spacing, and adequate plant nutrition encourage dense canopies that lead to developing conditions of high humidity. Fungus and infection thrive at temperatures ranging between 15 and 25 °C [17,45]. Additionally, the risk of disease transmission increases in plantations irrigated with overhead sprinklers as these methods amplify canopy moisture.

Sclerotial development, survival, and germination are also affected by temperature and moisture, either independently or in conjunction with other factors. Sclerotia germinate myceliogenically when the soil is wet and cool due to prolonged rains, irrigation events, and soil shading caused by crop canopy closure. Wet and cold soils (4–18 °C) are also ideal for ascospore production; however, the precise temperature range varies with the geographical basis of the *Sclerotinia* isolates [17,42]. After 2–6 weeks at 15 °C, sclerotia in the topsoil, which have been cold-conditioned, usually begin to generate apothecia [17]. This typically happens after a long period of rain or irrigation when the soil surface is slowed in drying. Prolonged leaf wetness (16–48 h) and temperatures ranging from 12 °C to 24 °C facilitate the germination of ascospores and subsequent infection [43]. With sufficient water availability, mycelia can spread from colonized senescent tissues into the robust host tissues of leaves, stems, buds, pods, and other plant parts. Maximum sclerotial development occurs at temperatures above 25 °C, with moderate development observed below 20 °C. However, sclerotial formation is significantly inhibited at temperatures below 20 °C [2].

## 8. Infection Process and Mechanisms

As a necrotrophic parasite, *S. sclerotiorum* kills host cells before advancing mycelium. The fungus produces oxalic acid necessary for effective pathogenicity and white mold disease development. Oxalic acid is poisonous to the host tissue and binds calcium in the intermediate lamellae, compromising plant tissue structural integrity [46]. The drop in extracellular pH stimulates the release of cell wall-disintegrating enzymes. The fungus releases small effector proteins that neutralize host defense responses and promote *S. sclerotinia* infection. These suggest that *S. sclerotiorum* employs a diverse arsenal to cause infection in its host. 

Using *Brassica napus* and *S. sclerotiorum* as model organisms, several transcriptome investigations have uncovered the underlying molecular and physiological events of host-pathogen interactions. To comprehend the differential defense response to *S. sclerotiorum* in a susceptible and resistant *B. napus* line, Wu et al. [47] employed transcriptome analysis to categorize 13,313 genes into functional groups and examined the expression levels of genes with hydrolase-related functions. In the resistant line, 9001 genes were differentially expressed compared to the susceptible line. Key differences in susceptibility and resistance were linked to the degree of gene expression changes involved in pathogen recognition, jasmonic acid/ethylene signaling pathways, WRKY transcription regulation, MAPK signaling, and the biosynthesis of indolic glucosinolate and defense-related proteins. 

Seifbarghi et al. [48] utilized RNA-sequencing (RNA-seq) to catalog genes expressed and upregulated during *B. napus* infections, focusing on early events. Many genes involved in polysaccharide degradation, including *SSPG1*, *SSPG3*, and *exoPG1*, showed significant expression levels at 24- and 48 h post-inoculation (hpi). Further studies by Chittem et al. [49] revealed global transcriptional alterations in S. sclerotiorum during the infection of rapeseed plants with varying susceptibilities to the pathogen. The roles of peroxisome-related pathways, cell-wall disintegration, and host metabolite detoxification have been found as the fundamental mechanisms underlying the pathogenesis of *S. sclerotiorum* in *B. napus*. At 8–16 and 24–48 hpi, 1301 and 1214 genes in *S. sclerotiorum* were differentially expressed during infection of the susceptible line, respectively. Meanwhile, 1311 and 1335 genes were differentially expressed when infecting the resistant line at the same time points, respectively. Up-regulated gene sets in both rapeseed lines showed considerable enrichment for gene ontology categories related to cell wall disintegration, detoxification of host metabolites, fatty acid ß-oxidation, the glyoxylate cycle, and oxidoreductase activity. Effector genes associated with host cell death showed elevated expression during the late stage of infection, while the expression of effector genes linked to host defense was repressed during the early infection stage [49]. 

The alterations in gene expression and associated pathways in both *B. napus* and *S. sclerotiorum* during different phases of infection were also studied [50]. At 6, 24, and 48 hpi, a total of 1986, 2217, and 16,079 genes were differentially expressed, respectively, in *B. napus*, while in *S. sclerotiorum* the corresponding numbers were 1511, 1208, and 2051, respectively. At 24 hpi, salicylic acid was activated while the jasmonic acid pathway was inhibited. Furthermore, after 24 hpi, the expression of most genes encoding cell wall-degrading enzymes in *S. sclerotiorum* and hydrolytic enzymes in *B. napus* was augmented, which coincided with the appearance of leaf necrosis [50]. It is anticipated that *S. sclerotiorum* stimulates salicylic acid synthesis in plants during early-stage infections to encourage infection and that following a successful *S. sclerotiorum* infection, multiple cell wall disintegrating enzymes are generated to consume nutrients [51].

## 9. Narrow Genetic Structures of the Pathogen

A combination of mycelial compatibility group (MCG) [52], inter-simple sequence repeat (ISSR) [53], random amplified polymorphic DNA (RAPD) [54], RFLP [55], sequence-related amplified polymorphism (SRAP) [56], simple sequence repeat (SSR) [57,58], and universal rice primers (URP) [59] techniques have been frequently utilized to examine the genetic structure of *S. sclerotiorum* isolated from various areas and hosts. Several of these population genetic analyses have found that the genetic makeup of *S. sclerotiorum* is clonal and highly conserved, with a single or small set of multilocus genotypes (MLGs) dominating the population globally and temporally [1,60]. The evidence of clonality is consistent with homothallic mating (self-fertilized sexual reproduction) and extensive asexual reproduction by sclerotia. Population surveys of crops like soybeans, cabbage (*Brassica oleracea*), canola, and kiwifruit (*Actinidia deliciosa*) in New Zealand, Australia, Europe, and North America have provided substantial support for this assumption [61,62]. On the contrary, many studies have an abundance of genotypic variation, a small clonal fraction, and a seemingly random connection of marker alleles [53,59]. These investigations have documented a lack of heterogeneity across geographic populations. Moreover, there are no conclusive indications of host specialization among *S. sclerotiorum* isolates [52]. These imply that outcrossing or recombination occurs irregularly in the *S. sclerotiorum* life cycle, which aids in shaping its population profile in the investigated areas [63].

The mating system is one of the primary evolutionary forces contributing to the formation of genetic structure. Isolates of heterothallic species typically carry one of two variants of the mating-type antigen (*MAT*). Only isolates with *MAT* differences are able to reproduce sexually. Homothallic (self-compatible) species carry both versions of *MAT* in a single nucleus, generally closely linked or fused [64]. The two variants of *MAT* in heterothallic are idiomorphs rather than alleles because they lack overall DNA sequence similarity. The *MAT* loci, idiomorphs, and genes are named after transcription factor-encoding alpha1 and high mobility group (HMG) domain genes [65]. *S. sclerotiorum* is an ideal example of a homothallic organism. There is evidence of both selfing and outcrossing and the fusion of *MAT1-1* and *MAT1-2* idiomorphs at the *MAT* locus [66]. Four genes make up the *MAT* of *S. sclerotiorum*. Isolates with the *MAT1-1* type have the genes *MAT1-1-1* and *MAT1-1-5*. Likewise, isolates with the *MAT1-2* pattern contain the *MAT1-2-1* and *MAT1-2-4* genes. Identical genes are present in the closely related species *B. cinerea* but are organized in a heterothallic *MAT* arrangement. There are instances where mating type changes from heterothallism to homothallism [67] and vice versa [68] in a single generation. Over longer evolutionary timescales, mating systems tend to diversify, and homothallic species are believed to have descended from heterothallic progenitors [69]. The molecular evidence for mating-type shifting does not rule out the possibility of an inversion being involved. The two *MAT* alleles in *S. sclerotiorum* have different orientations in the 3.6 kb region and undergo inversion in each meiotic generation. The inversion of MAT causes the flipping of two genes and the truncation of one gene, which is linked with changes in the expression of the *MAT* gene [70]. It is essential to determine if mating type shift in other filamentous ascomycetes is caused by a MAT inversion comparable to that of *S. sclerotiorum*.

## 10. Early Detection of White Mold

Early detection of white mold may lead to better disease management outcomes. Early identification prevents disease progression and lowers the risk of consequences. However, disease detection, diagnosis, and quantification must be inexpensive and reliable to enhance disease control tactics, assist producers in making management decisions, and maximize net profitability. Molecular and sensor-based techniques can assist growers in the timely detection of white mold disease.

### 10.1. Molecular Diagnosis of White Mold

With the development of molecular marker-based tools and techniques, it has become simpler and easier to identify, characterize, and detect plant pathogens that were previously difficult and time-consuming to confirm using classical visual-based inspection methods. A number of molecular approaches have demonstrated great sensitivity and specificity in detecting pathogens in a relatively short period of time using small amounts of DNA and/or RNA. Restriction fragment length polymorphisms (RFLP), sequence divergence within the nrDNA region (ITS, IGS), 28S nrRNA region (LSU), glyceraldehyde 3-phosphate dehydrogenase (GAPDH), β-tubulin, heat shock protein (HSP60), calmodulin (CAL), and *ras* protein, existence or nonappearance of group I introns in the nuclear small-subunit (nSSU) of the rDNA, single-nucleotide polymorphisms (SNPs) in the genome, and segregatory multiplex PCR [71,72,73] have accelerated the identification and characterization of *S. sclerotiorum* (Table 2). Typical PCR-based objects for identifying the fungus are the sequences of the ITS region (ITS1, 5.8S gene, ITS2) of ribosomal DNA (rDNA) [72]. However, the ITS region is nearly identical within the Sclerotiniaceae and almost unsuitable for species identification [74]. PCR amplification of other gene loci, such as GAPDH, HSP60, DNA-dependent RNA polymerase subunit II, β-tubulin, CAL, or laccase2 (*lcc2*), have been used to identify species within Sclerotiniaceae [72,75]. Multiplex PCR with *lacc2*, CAL, and elongation factor-1 alpha gene was able to simultaneously distinguish *S. sclerotiorum* and other closely related species in complex microbiota [76]. SNPs are among the most pervasive types of genetic variation utilized to identify *S. sclerotiorum* [77] and explore intra- and inter-species differentiation [72]. The SNP-based protocol appears more time-consuming and expensive than multiplex PCR.

**Table 2:** Standard molecular diagnostic markers used to detect *Sclerotinia sclerotiorum* with relative application and advantages.

Methods	Application	Advantage	References
Restriction fragment length polymorphisms (RFLP)	Comparing relatedness and detecting intra- and inter-specific variants in the genus	Quick, simple, cost-effective, and highly reproducible	[29]
DNA barcoding	Species-level identifications based on DNA sequences from a signature region of the genome	Sequence availability and high accuracy	[38,72]
Simplex PCR	Single species identification based on the presence of marker gene	Higher sensitivity and accurate detection	[76]
Single-nucleotide polymorphisms (SNP)	Detect genetic variants (mutations) within Sclerotinia populations using PCR amplification and oligonucleotide hybridizations	Fast and robust and is amenable to the high-throughput screening of samples	[77]
qPCR	Identification and quantification of the pathogen in plant infections	A rapid, accurate, and reliable detection; and quantification of very low amounts of pathogen DNA	[78]
Multiplex qPCR	Multiple species identification in plant material, including seeds using species-specific primers	Cost-effective, time-saving, and higher throughput	[73]
Seed-soaking-based PCR	*S. sclerotiorum* detection in seeds or plant components without prior fungal DNA extraction	Precise detection and reduced assay time (about 9 h)	[79]
Loop-mediated isothermal amplification (LAMP)	Fungal, plant, and soil samples	Sensitive, specific, rapid, and suitable for field application	[9]

The rapid and precise detection and quantification of *S. sclerotiorum* are facilitated by real-time quantitative polymerase chain reaction (qPCR) [78]. Due to its great sensitivity and the utilization of fluorescent chemistry, the qPCR permits the detection of minimal amounts of pathogen DNA during an asymptomatic latent infection or at the onset of a disease [80]. The multiplex qPCR simultaneously detects and quantifies the seed-borne inoculum of *S. sclerotiorum* and other fungi [73]. Seed-borne inoculum of *S. sclerotiorum* can also be quickly detected at extremely low incidence levels (0.5%) using a seed-soaking-based PCR approach that does not require fungal DNA extraction [79]. In addition, the technique is highly specific to *S. sclerotiorum*, with no other fungi being detected incorrectly, thereby avoiding the difficulties associated with misidentification due to saprophytic fungi contamination. A new gene amplification approach, loop-mediated isothermal amplification (LAMP), has recently been employed to synthesize larger quantities of *S. sclerotiorum* DNA and give a visible byproduct in the absence of thermal cycling. The real-time quantitative LAMP (Ss-qLAMP) assay or a calcein ion indicator-based LAMP (Ss-cLAMP) assay detected *S. sclerotiorum* in soil and soybean (*Glycine max*) tissues without needing DNA extraction [9]. The LAMP technique is regarded as a sensitive, time-efficient, and field-applicable method for rapid pathogen identification.

### 10.2. Sensor-Based Detection of White Mold Disease

Sensor devices are promising tools for the automated detection, diagnosis, and quantification of plant diseases. Sensors employed for plant disease identification are broadly classified into optical and non-optical categories. Several types of optical sensors are known, including multi- and hyperspectral reflectance, RGB, light detection and ranging (LiDAR), fluorescence, and thermal sensors. Infrared thermography and spectral reflectance sensors are particularly versatile, operating across ground, air, and satellite platforms. This makes them ideal for plant disease detection and phenotyping. While remote sensing with airborne platforms is beneficial for finding soilborne disease patches in the field or at later stages of disease outbreaks, proximal sensing platforms are suited for disease detection at the leaf and plant scale [81]. The most popular non-optical sensors for disease detection can perceive volatile organic compounds (VOCs) of plants, pathogens, or both during disease progression. Non-optical devices include electronic noses (e-nose), gas chromatography-mass spectrometry (GC-MS)-coupled VOC-trapping devices, and other cutting-edge VOC sensors [10].

Various sensors have been exploited to detect white mold disease on aerial plant parts. An indoor UAV (Unmanned Aerial Vehicle) low-altitude remote sensing simulation platform equipped with multi-sensors was used to identify white mold disease on *B. napus* leaves [11]. The study assessed the detection performance of four machine learning models, such as random forest (RF), K-nearest neighbor (KNN), support vector machine (SVM), and naïve Bayes (NB), using thermal pictures and fused images. The multi-sensor fusion significantly improved classification accuracy and reliably detected *S. sclerotiorum* on oilseed rape. 

Several studies explored hyperspectral imaging to detect *S. sclerotiorum* disease on leaves and stems [82,83]. When combined with chemometrics, hyperspectral imaging demonstrated high efficiency for *S. sclerotiorum* disease identification. Furthermore, Shoute et al. [84] developed an impedimetric non-Faradaic biosensor to detect the binding of ascospores utilizing an interdigitated electrode and anti-*S. sclerotiorum* polyclonal antibodies as probes. More recently, an optical features-based new approach was developed for detecting white mold disease [85]. Using linear discriminant analysis (LDA) and SVM models, the method achieved 100% classification accuracy in distinguishing infected leaves from healthy ones. 

The development of a mobile, automated tool that can be set up on-site to detect and measure the presence of minute amounts of ascospores in the air and works as a component of a network of systems for epidemic forecasting is highly useful. This type of ascospore detection biosensor offers significant prospects for advancing disease detection capabilities. However, despite the rapid growth in research on sensor technologies for plant disease detection, further advancements are required to identify and detect disease-specific signatures. These improvements are crucial before such techniques can be effectively implemented as practical tools for disease identification and site-specific management in agricultural fields. 

## 11. Conventional to Non-Conventional Disease Management

**Figure 5:**
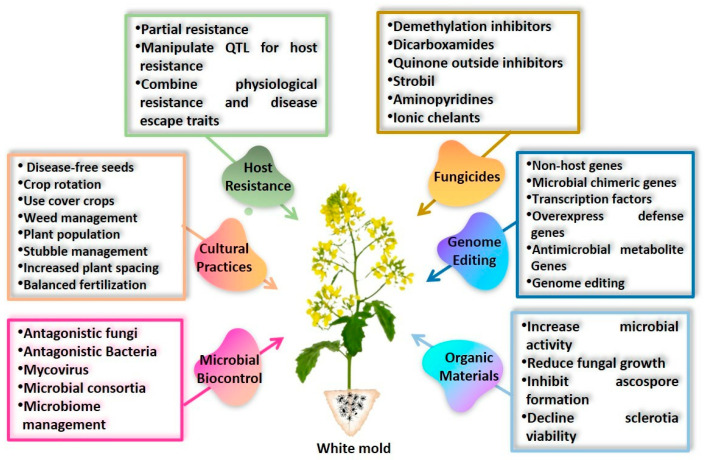
Common conventional and non-conventional strategies to control white mold diseases in plants. These strategies include cultural practices, biological controls using beneficial microbes and organic amendments, chemical treatments with fungicides, and genetic resistance using breeding and genome editing. Each strategy involves a number of practices to reduce disease incidence and improve crop health.

White mold caused by *S. sclerotiorum* is challenging to control due to the unusual ability of sclerotia to persist for prolonged durations and the formation of both soil-borne and air-borne inoculum. Although the fungus can infect plants at any time during crop development, *S. sclerotiorum* management necessitates vigilant attention throughout the growth cycle. However, a single control method is hardly adequate to manage the disease successfully. An effective approach often requires a combination of several methods. Below are described various cultural, biological, physical, and chemical approaches for making the crop microclimate less conducive to infection, using efficient fungicides to protect sensitive plants, eliminating sources of inoculum, and manipulating the host plant’s resistance to the disease (Figure 5).

### 11.1. Modifying Crop Microclimate by Cultural Practices

Management of white mold disease relies heavily on cultural and agronomic practices due to the unavailability of resistant cultivars and the broad host range. Reducing plant populations, rotating crops to non-hosts, increasing row width, modifying tillage practices, and planting cover crops are key cultural management practices that make the crop microclimate less conducive for the pathogen, thereby lessening the risk of disease development [17]. Since repeated cultivation of susceptible crops helps increase sclerotial populations in each successive crop, weed management and appropriate crop rotation are crucial to decrease the sclerotial load in the soil. A minimum 2–3-year crop rotation is essential where suitable non-host crops should be included [86]. Ascospore production and its dissemination can also be slowed through crop rotation and reduced plant density. Corn, wheat, barley, oats, and sorghum are all examples of small grain crops that are resistant to *Sclerotinia* spp. Hence, these crops make good rotation crops. If the possibility remains of colonizing these grain crops by an aggressive *Sclerotinia* strain, avoiding contaminated or neighboring areas for 1–4 years may be the most effective management strategy if it is economically feasible [87]. Nevertheless, the sclerotia number in the soil may need to be reduced by pausing cultivation for two to three years [17]. 

Increasing airflow and decreasing humidity in the crop canopy can be accomplished through canopy management practices such as increasing row spacing or decreasing seeding rates. Numerous crop-specific studies have revealed that improved airflow through plant canopy helps reduce the occurrence of apothecia, ascospore production, and, thereby, white mold disease [88]. Over-fertilizing and early planting can lead to tall and bulky crops at blooming, creating denser canopies and placing crops at risk of disease in locations with heavy rainfall [89]. Therefore, applying balanced fertilizers and late planting can be crucial. 

Managing stubble to reduce the survival of viable sclerotia from one growing season to the next has proven useful. Some of these practices include burning crop residues or using irrigation to speed up the breakdown of sclerotia. No-till systems can also help reduce disease incidence [90] because sclerotia remain on the soil surface, making them more susceptible to destruction from predators, drying, and ultraviolet light [17]. 

Contaminated seeds pose a significant risk. Seeds contaminated with mycelia or sclerotia have the potential for long-distance dissemination of the fungus [91]. Moreover, *S. sclerotiorum* uses the seeds as a nutrient source to form sclerotia in soil [92]. Sowing such seeds allows the pathogen to colonize new fields. As a result, saving seeds from infected crops for replanting is not recommended to reduce the risk of the disease spreading to healthy areas.

### 11.2. Use of Natural Resistant Sources

The most cost-effective and long-term strategy for managing the disease is to plant resistant varieties. Various screening approaches have identified complete [93] and partial resistance [94], including sclerotia-resistant traits for partial stem resistance [95]. While no commercial cultivars of most crops are fully resistant to *S. sclerotiorum*, a few show slight resistance [44]. Efforts are being made to develop prime cultivars resistant to *S. sclerotiorum* and *S. minor* that can thrive in diverse environments [96]. Several forage and grain legumes are resistant to *S. trifoliorum*, including alfalfa, clover, and faba beans [97,98]. However, commercially available resistant cultivars for these crops remain unavailable, though breeding programs are actively working toward this goal. Notably, incorporating *S. sclerotiorum* resistance from wild *Cicer* populations into commercial chickpea varieties has demonstrated modest stem resistance [99].

To lessen the danger of yield losses and inoculum load in forthcoming seasons, using partially resistant cultivars is recommended for crops susceptible to *Sclerotinia* species when such varieties are available [100]. Disease management through improved host resistance requires both conventional and molecular breeding to accelerate the variety development process. Resistance to sclerotia is polygenic and controlled by a complex interplay of many low-effect genes [101]. Breeding resistant varieties is challenging and costly when several minor genes influence *Sclerotinia* tolerance in multiple species. Additionally, there is a risk of introducing undesirable traits through linkage drag during breeding processes. Consequently, plants in natural populations display a spectrum of resistance, from full susceptibility to varying degrees of resistance, a phenomenon referred to as quantitative disease resistance. 

Early efforts to identify resistance genes against *Sclerotinia* utilized quantitative trait loci (QTL) mapping. For example, Gyawali et al. [102] conducted a genome-wide association study (GWAS) on global *B. napus* germplasm, identifying 669 polymorphic loci associated with resistance to *S. sclerotiorum*. The *S. sclerotiorum* resistance was linked to 21 alleles, while the susceptibility was linked to 13 alleles. Using 347 markers and 35 QTLs linked to disease resistance, white mold resistance in *B. napus* was mapped [103]. A GWAS pinpointed 103 QTLs in soybean, which were subsequently mapped to 11 and 16 loci linked with greenhouse and field resistance, respectively [104].

Both canola and soybean have *Sclerotinia* resistance, but neither contributes much to phenotypic variance, usually accounting for less than 10% [94,104]. As a result, researchers are exploring alternative approaches, such as GWAS mapping conducted with wild plant populations. Advances in high-throughput sequencing, omics technologies, data analytics, and an improved understanding of plant defense mechanisms have enabled the manipulation of QTLs and host genes to enhance *Sclerotinia* resistance and reduce susceptibility. 

Resistance mechanisms against *Sclerotinia*, as described by Ding et al. [105], involve triggering defense signaling pathways, producing secondary metabolites that inhibit *Sclerotinia* infection, or generating antimicrobial peptides, proteins, and enzymes that affect the integrity of cell walls or neutralize pathogenicity factors. According to the findings of Wang et al. [101], the vast majority of genes *in B. napus* implicated in downstream defense responses, such as secondary metabolite enzymes, reactive oxygen species (ROS) generation, detoxification, and oxidative protection, are linked to *Sclerotinia* resistance. This is the case regardless of whether the genes are located in the nucleus or the cytoplasm. It is essential to consider a diverse range of cellular pathways when breeding crop types resistant to *Sclerotinia*. There have been reports of QTLs linked to physiological resistance (such as phytoalexin production) to *S. sclerotiorum*. It is hypothesized that a combination of physiological resistance and disease escape traits (lodging resistance, open canopy architecture, and maturity timing) can reduce the severity of *S. sclerotiorum* disease [106]. However, this may complicate attempts to create varieties resistant to *S. sclerotiorum*, as traits associated with these disease resistance mechanisms may not be easily breedable.

### 11.3. Use of Transgenes

Classical breeding is challenging because most crops lack strong, large-scale resistance. Hence, targeted genetic modification or genetic engineering for solid resistance to *S. sclerotiorum* is considered a cost-effective choice. There are many examples where various plant species have been transformed with genes from heterogeneous sources that show enhanced resistance to *S. sclerotiorum*. Canola transformed with the *Raphanus sativus* defensin gene and *Trichoderma atroviride* chimeric *chit42* gene showed improved *S. sclerotiorum* resistance and lower sclerotinia stem rot disease [107]. Overexpression of *Arachis stenosperma* (*AsTIR19*) truncated NLR (TNx) gene in tobacco plants significantly reduced infection by *S. sclerotiorum* [108]. The resistance of many crops to *S. sclerotiorum* has been boosted by the overexpression of oxalate oxidase genes [109,110]. Improving plant resistance to infections typically involves the induction of defense signaling pathways (Salicylic acid, jasmonate, and ethylene), *PRs* (pathogenesis-related proteins), and upregulation of the expression of defensive response master switches, such as mitogen-activated protein kinases (MPKs) or transcription factors [108,111]. *Arabidopsis* and canola plants are more resistant to *Sclerotinia* when WRKY transcription factors and/or MPKs are overexpressed [112,113]. Overexpression of downstream defense genes, such as polygalacturonase-suppressing proteins or chitinase genes, is another strategy for retarding disease progression [114]. Recent research has demonstrated that plant antifungal metabolites can inhibit *Sclerotinia*, suggesting that their expression could be altered to increase resistance. The antifungal effectiveness of metabolites extracted from improved-resistant soybean stems has recently been reported [115]. The metabolite extracts disrupt *S. sclerotiorum* enzymes involved in fungal sterol and lipid production, hence inhibiting the growth of the pathogen.

### 11.4. Genome Editing

Genome editing is the foundation of new breeding techniques to improve plant resistance to the pathogen. Currently, draft sequences are available for the vast majority of crop genomes. In this context, even in crops with very complex genomes, multiple types of precision genome editing can be applied to alter all target gene alleles simultaneously to achieve a desired phenotype. The commonly used genome editing tools include oligo-directed mutagenesis (ODM) and the use of sequence-specific nucleases (SSNs) such as zinc-finger nucleases (ZFN), transcription-activator-like effector nucleases (TALEN), and clustered regularly interspaced short palindromic repeats/CRISPR-associated protein (CRISPR/Cas9). By replacing random mutagenesis with targeted mutations, these genome editing technologies significantly reduce the frequency of unintended changes. TALENs can specifically target a given genomic location. However, programming them at the amino acid level is time-consuming, and releasing them jointly into the crop nucleus is challenging [116]. On the contrary, genome editing using the bacterial CRISPR/Cas system in eukaryotic cells is simple to perform, inexpensive, and compatible with multiplexing [117]. Genome editing with CRISPR/Cas does not need protein engineering, effective target cleavage, or the use of large protein molecules, in contrast to TALENs or ZFNs. CRISPR/Cas has already revolutionized the history of genome editing through highly feasible multi-target site cleavage and DNA-free delivery of the CRISPR/Cas construct, which can be carried out by conveying preassembled Cas protein and guiding RNA into the cells of the target organism [118]. As a result, TALENs or ZFNs have quickly been surpassed by CRISPR/Cas9 as the most common genome editing technique.

**Figure 6:**
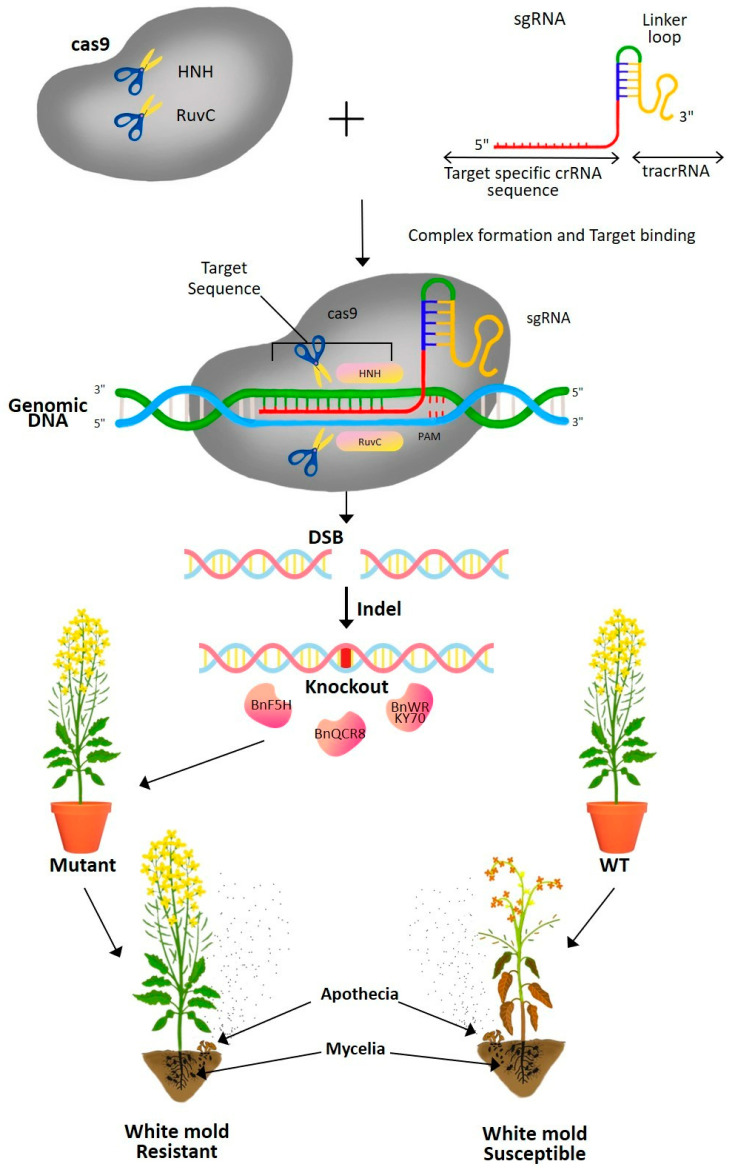
Schematic diagram showing genome editing of rapeseed (*Brassica napus*) plants through CRISPR-Cas9 technology for generating white mold-resistant plants. The Cas9 enzyme, guided by a specific sgRNA (single guide RNA), targets a specific genomic DNA sequence to create a double-strand break (DSB). The repair process through non-homologous end joining (NHEJ) results in gene knockout or insertion/deletion (Indel) mutations, leading to altered susceptibility to white mold in mutant plants compared to wild-type (WT) plants. The resistant mutants exhibit reduced infection and disease symptoms when exposed to white mold pathogens. (HNH, an endonuclease domain named for characteristic histidine and asparagine residues; RuvC, an endonuclease domain named for an *Escherichia coli* protein involved in DNA repair; crRNA, CRISPR RNA; tracrRNA, trans-activating crRNA; sgRNA, single guide RNA; PAM, Protospacer-Adjacent Motif; Indel, insertion and/or deletion).

The CRISPR/Cas9 system consists of two components: the Cas protein, which typically initiates a double-strand break (DSB) in the DNA, and the single guide RNA (sgRNA), which directs the Cas protein to the genomic target region of interest (Figure 6) [119]. A predesigned 20-nt changeable guide sequence within the sgRNA complementary to the target sequence provides the system’s specificity. Cas9, a protein derived from the gram-positive bacterium *Streptococcus pyogenes*, is the most frequently employed Cas protein [118,119]. If the entire genome sequence is known, it is possible to construct a highly specific sgRNA by choosing a 20 bp sequence, which is highly conserved in all alleles of a single gene of interest and distinct from the remaining genomic sequence. The formation of DSBs in the target DNA activates either the error-prone nonhomologous end joining (NHEJ) or direct homologous recombination (DHR) DNA repair mechanisms [119]. This DNA repair process may result in gene knockouts, genomic modifications, or random gene insertions, leading to the development of disease-resistant mutant plants.

Meanwhile, specific CRISPR/Cas systems to improve crop resistance against *S. sclerotiorum* have been described. *Sclerotinia* effector-like protein SsSSVP1 targets a plant gene during infection [120]. Zhang et al. [121] reported identifying eight homologous copies of that gene in *B. napus*. Mutants of *B. napus* with one or more modified copies of BnQCR8 were shown to be more resistant to *S. sclerotiorum* after reducing the number of BnQCR8 copies using CRISPR/Cas9 [121]. Determining which host gene(s) is the best target of *Sclerotinia* effectors or which transgenes would be the most effective is a challenge in targeted genetic manipulation strategies.

### 11.5. Protection of Susceptible Plants by Fungicides

*Sclerotinia* is commonly managed using a combination of fungicides and cultural practices. Fungicides, which are used to prevent losses due to disease, come in a wide range of modes of action and are widely accessible for purchase worldwide. However, in some regions, the number of fungicide applications per season is capped, and not all fungicides are approved for use on every crop. Moreover, many fungicides lack systemic activity, rendering them ineffective in protecting untreated parts of the plant [17]. Demethylation inhibitors (DMIs), dicarboxamides, quinone outside inhibitors (QoIs), and strobil are among the best fungicides for controlling *S. sclerotiorum* (Table 3) [17,20]. On the other hand, for managing *Sclerotinia minor* on peanuts, recommended fungicides include aminopyridines, dicarboxamides, selective contact fungicides, and ionic chelants [100]. Many of these fungicides are prophylactic and must be applied before an infection occurs. If applied after symptom onset, their effectiveness is significantly reduced. While foliar fungicides can prevent damage when applied prior to symptom development, they are generally more effective as preventative measures rather than curative treatments [17].

The early flowering stage is commonly chosen to apply fungicides since plants are more vulnerable to *Sclerotinia infection* at this time. However, the most cost-effective time to treat plants with fungicides is during the seasons with moderate to high infection risk [20,44]. Fungicide application during this time aims to ensure the protection of early blooms, maximum chemical penetration into the canopy, and prevention of pathogen entry points. In areas with elevated disease pressures or extended flowering times, a second application is frequently directed at later phases of blossoming [122]. Even though fungicide spraying is associated with high cost, single preventative fungicide spraying is neither practical nor desirable during bloom due to the unpredictable nature of the disease (prevalence varies). In crops like canola, which flowers for as long as six weeks in some countries like Australia, preventative fungicides remain effective for as long as three weeks; thus, multiple doses of fungicide are often necessary. Despite receiving two foliar fungicide treatments, high infection levels have still been observed in some cases. These clearly show how challenging it is to control the disease and how crucial it is to understand the environmental triggers for more targeted care.

In leafy vegetables like lettuce, the timing of fungicide application is particularly critical. Predictive models have been developed to anticipate disease onset and enable more precise interventions [43]. About 80–96% decrease in *Sclerotinia* lettuce drop disease has been shown to be achieved with strategic plant-targeted dicarboxamide spraying [123]. Moreover, dicarboxamides are more effective than other licensed fungicide classes in lettuce when the disease pressure is high [123]. To prevent lower leaf infections and slow mycelial growth, it is essential to drench soil immediately after transplanting and administer fungicides before canopy closure. However, the increasing demand for vegetable production with minimal pesticide inputs, combined with limited legal fungicide options in some countries, poses serious challenges. Hence, fungicide use decisions must balance the immediate benefits of protecting the current harvest with the long-term goal of reducing the soil sclerotia load for future crops. Economic considerations also weigh heavily, as growers must assess whether the benefits of fungicide application justify the associated costs.

Fungicide use presents several other challenges, including the risk of resistance development. Excessive use of a single type of fungicide can result in the emergence of fungicide-resistant pathogen populations. This is largely because most key fungicides depend on a single mode of action, increasing the likelihood of mutations at specific genomic sites in the pathogen, which can ultimately lead to resistance development [20]. However, *S. sclerotiorum* demonstrates a limited predisposition for fungicide resistance, as it is homothallic and predominantly reproduces asexually [124]. Nevertheless, decreased sensitivity to SDHIs has been reported in some strains in France, alongside global cases of resistance to MBC and dicarboxamides [125]. Therefore, to effectively manage *S. sclerotiorum*, it is crucial to implement diverse strategies rather than relying exclusively on fungicides. Furthermore, the selective pressure from fungicide use can also promote resistance in non-target pathogen populations, complicating disease management efforts.

### 11.6. Eliminating the Pathogen by Biological Control Agents

Due to the serious risks connected with fungicide sprays and the difficulty of existing strategies, biological control of white mold disease has gained interest in eliminating the pathogen. Biological controls limit dependency on chemicals and the rise in fungicide-resistant strains in pathogen populations while lessening both environmental and health risks. Biocontrol agents are typically less effective than synthetic pesticides because of their sensitivity to various biotic and abiotic factors. Yet, safer biological approaches are being pursued because of the many positive benefits.

#### 11.6.1. Application of Solo Microbial Agent

Numerous fungal and bacterial strains have been explored as possible biocontrol agents for *S. sclerotinia. Coniothyrium*, *Trichoderma*, *Bacillus*, *Pseudomonas*, *Streptomyces*, and *Serratia* are some of the many fungal and bacterial genera that have been studied [42,126,127,128,129]. *C. minitans*, a fungus that preys on other fungi, can attack and dissolve soil sclerotia [130]. Apothecia formation and ascospore production, necessary for the onset of disease symptoms, are inhibited by *C. minitans* during its attack on *Sclerotia* [126]. The application of *C. minitans* has shown evidence of effectiveness in evading white mold disease in dry beans, lettuce, carrots, alfalfa, and soybeans (Table 4). The application of *C. minitans* reduced *S. sclerotiorum* disease symptoms by 10–70% in field experiments across multiple crops [42], while sclerotia formation was reduced by up to 95% in certain trials. Additionally, applications of Contans WG, a commercially available *C. minitans* biocontrol product, in combination with a dicarboximide fungicide in a low dose, prevented the appearance of *S. sclerotiorum* symptoms in infected bean plants (*Phaseolus vulgaris* L.) during greenhouse trials [131]. This highlights the opportunity for the Contans WG to be included in an integrated disease management (IDM) system against *S. sclerotiorum*. Li et al. [132] demonstrated that three administrations of *C. minitans* conidia at a concentration of 5 × 10^6^ mL^−1^ to alfalfa blooms effectively prevented sclerotinia pod rot under field conditions. Similarly, spraying bean plants with a *C. minitans* spore suspension at the flowering stage minimized the white mold occurrence by 56% [133]. Incorporating *C. minitans* into the topsoil before soybean sowing resulted in a 68% decrease in the disease severity index and a 95.3% decrease in the sclerotia number in the soil [134]. Despite numerous accounts of *C. minitans* successfully controlling *Sclerotinia*, its effectiveness in the field remains inconsistent [135]. These variations may be attributed to the diverse and variable soil conditions encountered in field environments [135,136].

**Table 4:** Fungal and bacterial agents with mycoparasitic and antagonistic activity against *S. sclerotiorum*.

Species	Host Plants	Disease	References
*Coniothyrium minitans*	Dry bean	White mold	[133]
*C. minitans*	Lettuce	Sclerotinia disease	[137]
*C. minitans*	Carrot	Sclerotinia rot	[138]
*C. minitans (Contans)*	Lettuce	Lettuce drop	[139]
*C. minitans* CON/M/91-08 (Contans^®^WG) *Streptomyces lydicus* WYEC 108 (Actinovate^®^AG) *Trichoderma harzianum* T-22 (PlantShield^®^HC),	Soybean	Sclerotinia stem rot	[134]
*C. minitans LU112* *T. virens LU556*	Cabbage	Sclerotinia disease	[140]
*Fusarium oxysporum* (S6)	Soybean	Sclerotinia disease	[141]
*Coniothyrium minitans* *Microsphaeropsis ochracea*	NT	-	[142]
*Sporidesmium sclerotivorum*	Soybean	Sclerotinia stem rot	[143]
*Trichoderma asperellum*	Common bean	White mold	[144]
*Trichoderma asperellum* T2 *Trichoderma hamatum* T3 *T. harzianum* T6	Arabidopsis	Root rot	[145]
*C. minitans* *T. atroviride*	Alfalfa	sclerotinia blossom blight	[132]
*T. harzianum-8* *T. atroviride PTCC5220* *T. longibrachiatum PTCC5140*	Canola	Stem rot	[146]
*Ulocladium atrum*	Canola	Sclerotinia disease	[147]
*Bacillus subtilis* BY-2 *Bacillus megaterium* A6	Oilseed rape	Sclerotinia disease	[148]
*Bacillus cereus* SC-1	Canola	Stem rot	[149]
*Bacillus subtilis* SB01 *Bacillus subtilis* SB24	Soybean	Sclerotinia stem rot	[150]
*Bacillus cereus* *Bacillus amyloliquefaciens*	Carnation	Sclerotinia root rot	[151]
*Bacillus* sp. B19 *Bacillus* sp. P12 *Bacillus* amyloliquefaciens B1	Common bean	White mold	[152]
*Streptomyces lydicus* WYEC 108	*Brassica* vegetables	Sclerotinia disease	[89]
*Streptomyces exfoliates* FT05W *Streptomyces cyaneus* ZEA17I	Lettuce drop	Lettuce	[129]
*Serratia plymuthica* IC14	White mold	Cucumber	[126]

A number of *Trichoderma* strains restrict the hyphal development of *Sclerotinia*, and parasitize sclerotia, resulting in lower apothecia formation [153]. Geraldine et al. [144] conducted field studies with common beans over two years and found that applying *T. asperellum* decreased the frequency of *S. sclerotiorum* apothecia and the severity of the disease. Compared to the control group, the *Trichoderma* treatment benefited the quantity of pods produced by each plant and resulted in a yield increase of up to 40%. The colonization of sclerotia by *T. hamatum* decreased the apothecial abundance and caused carpogenic infection of cabbage in field experiments [154]. Under commercial greenhouse conditions, applying *T. harzianum* T39 reduced white mold on cucumber fruit and stems [155]. In a field-grown crop, soybean plants were protected from *S. sclerotiorum* by *T. harzianum* T-22, resulting in a 38.5% disease severity index reduction [134]. Approximately a hundred biofungicides and growth-promoting compounds derived from different *Trichoderma* spp. have been commercially available [153]. The use of *Trichoderma* spp. for biocontrol is still the subject of active investigation. Unfortunately, there is limited data on their efficacy against *Sclerotinia* in the field [42].

Many bacterial genera have shown the ability to control white mold disease. Several studies have been undertaken to prove that *Bacillus* can inhibit *Sclerotinia* in vitro [151,156]. Treating canola with *B. megaterium* (A6) and *B. subtilis* (BY2) as seed coating or as a spray during blooming led to the suppression of disease and considerable increases in crop yield in field experiments [148]. Two foliar sprays of *B. cereus* (SC1-1) protected canola against *S. sclerotiorum* in three field experiments over two seasons, achieving an efficacy level of 71–80% [149]. In field experiments, soybeans treated with strains (SB01 or SB24) of *B. subtilis* had significantly less severe disease [150]. Before transplanting, a root dipping in a bacterial suspension containing various *B. cereus* and *B. amyloliquefaciens* strains reduced *Sclerotinia* root rot in carnations by as much as 88% [151]. In pot trials, the severity of *S. sclerotiorum* disease on common bean and mustard plants was greatly reduced by adding several *Bacillus* [152]. While recommendations for commercial usage of *Bacillus*-based products appear promising, more field investigations are needed to prove their usefulness.

Actinobacteria, like *Streptomyces* spp., are essential members of the soil and rhizosphere communities. Due to their ability to produce an inclusive range of antimicrobial secondary metabolites, many *Streptomyces* are employed in plant disease management. These *Streptomyces* strains can colonize plant roots and form symbiotic links with plants, giving them attractive prospects for developing agricultural goods to combat disease and promote plant growth [157,158]. Notably, *S. griseoviridis* (K61) and *S. lydicus* (WYEC 108) are the active ingredients of the commercial biopesticide Mycostop (Verdera Oy, Espoo, Finland) and Actinovate (Novozymes BioAg Ltd., Saskatoon, SK, Canada). When applied to the soil, Actinovate has shown efficacy against *S. sclerotiorum* in *Brassica* vegetables [89]. In a field trial, soil drenching with Actinovate (*S. lydicus*) lowered the disease severity index by 30.8% and sclerotia number by 93.8% in harvested soybeans, in comparison to control plots [134]. A comparative performance study of three *Streptomyces* strains, *S. exfoliates* FT05W, *S. cyaneus* ZEA17I, and *S. lydicus* WYEC108, revealed that all three strains offered some level of protection against *S. sclerotiorum* infection in lettuce in the growth chamber [129]. Among these three strains, *S. lydicus* was the most effective. However, in the field, disease rates were lowered by *S. exfoliates* FT05W and *S. cyaneus* ZEA17I, but not by *S. lydicus* WYEC108. In addition to *Streptomyces*, other bacterial species show potential as biocontrol agents. For instance, *Serratia plymuthica* IC14 exhibited antifungal activity against *S. sclerotiorum*, preventing cucumber plants from developing white mold under greenhouse conditions [126]. Under greenhouse circumstances, this bacterium prevented cucumber from developing white mold caused by *S. sclerotiorum*. This highlights the importance of field-based evaluations for disease management strategies and suggests that diverse *Streptomyces* strains, alongside other bacteria, hold commercial potential as biocontrol solutions for managing white mold disease.

Mycoviruses have emerged as the new biocontrol agents of *Sclerotinia*. These viruses, such as single-stranded circular DNA viruses, single-stranded RNA, and double-stranded RNA, can replicate in sclerotia and use them as their hosts [159]. One of the DNA mycoviruses, such as SsHADV-1, or *S. sclerotiorum* hypovirulence-associated DNA virus 1, is capable of infecting *S. sclerotiorum* and conferring hypovirulence [160]. When SsHADV-1 viral particles were sprayed on *Arabidopsis thaliana* or *B. napus* leaves before inoculation with *S. sclerotiorum*, the plants showed less damage by the fungus. Further investigation found that this mycovirus converts pathogenic *S. sclerotiorum* into an endophyte by suppressing its key virulence genes or effector-like genes *Ss-Cmu1*, *SsITL*, and *SsSSVP1* [161]. In addition to affecting *Sclerotinia* gene expression, the virus ssHADV-1 infection of *S. sclerotiorum* helped regulate plant defenses. When the virus ssHADV-1-infects *Sclerotinia*-invaded plant tissues, it increases the expression of defense- and hormone-associated genes in *B. napus*, leading to increased plant growth. Even when hyphal fragments of an *S. sclerotiorum* strain infected with SsHADV-1 were sprayed on *B. napus* plants during early bloom, *Sclerotinia* stem rot severity was reduced by 30–67% [161].

#### 11.6.2. Microbial Consortia and Microbiome Management

Single-microbe inoculants are the standard for biocontrol agents on the market and in practice. However, there is increasing interest in utilizing groups of beneficial microbes to improve crop health [162]. Although studies on this approach are limited for managing *Sclerotinia*, they show promise in controlling the disease. For instance, pea plants demonstrated a lower mortality rate to *S. sclerotiorum* when inoculated with a combination of *T. harzianum*, *B. subtilis*, and *Pseudomonas aeruginosa* [163]. Similarly, a study in beans found that applying *T. viride*, *T. hamatum*, and *C. minitans* as soil drenches was more effective at preventing *S. sclerotiorum* infection than treatments with individual agents like *B. subtilis*, *Pseudomonas fluorescens*, or *S. griseoviridis* [164]. Notably, the highest level of disease suppression (a 100% reduction in disease severity) was achieved using either a combination of *T. hamatum* and *S. griseoviridis* or *C. minitans* and *S. griseoviridis*. These findings suggest that utilizing multiple biocontrol agents with complementary mechanisms of action may be more effective than single-agent treatments. 

The study of synergistic interactions between biocontrol organisms is still in its infancy and requires further research to unlock its full potential. The establishment of microbial consortia across various plant tissues and ensuring their stability throughout extended crop growth cycles, such as those typical of many row crops, remains a significant challenge for implementing measures to control *Sclerotinia* infections. Altering the microbiome of agricultural soil to encourage plant growth is an alternative strategy. It has been demonstrated that this approach can be feasible by implementing changes to farming practices (such as reducing reliance on agriculture and increasing the variety of crops grown) [165]. However, this is a relatively new field of research, and substantial knowledge gaps need to be filled before this strategy can be implemented.

#### 11.6.3. Organic Material Amendments

The incorporation of organic materials into soil is widely known to improve soil properties, plant health, and crop yield. Additionally, these materials can quantitatively and qualitatively alter soil bacterial and fungal communities by serving as a food source for soil microorganisms [166]. Increased microbial activity is often cited as a key reason organic amendments effectively suppress soil-borne fungal diseases [167]. Therefore, one effective strategy for managing *S. sclerotiorum* in contaminated soils is applying organic materials containing biologically active compounds [42]. Research by Huang et al. [168] reported that 46 of 87 organic amendments, when applied to the soil at a rate of 3% *w*/*w*, significantly reduced the growth of the fungus. However, at a lower application rate of 0.5% *w*/*w*, only three types of residues were effective. Amendments with high nitrogen content, such as fish meal, were the most successful in inhibiting ascospore formation. The authors suggested that the decline in soil sclerotia viability might be linked to the production of ammonia and ammonia-related compounds.

While organic soil amendments can enhance soil health, they may also have varying effects on the *S. sclerotiorum* population [169]. Some studies have shown that soils rich in organic matter promote the carpogenic germination of *S. sclerotiorum* sclerotia [170]. However, combining organic materials with antagonistic microorganisms has proven to be one of the most effective strategies for eliminating *Sclerotinia* from infested fields and minimizing the risk of pathogen multiplication. Huang et al. [168] found that sclerotia germination was reduced when the soil was amended with *C. minitans* or *Trichoderma virens*-infested organic residues. Moreover, Smolinska et al. [42] reported that the complete eradication of *S. sclerotiorum* sclerotia was achieved through the application of selected *Trichoderma* species on organic carriers made from agro-industrial wastes (potato pulp, wheat straw, strawberry pomaces, apple, dry onion rind, and rapeseed meal). *Trichoderma* overgrowth on plant residues inhibited the pathogen reproduction in these materials. Since mycoparasitic fungi rely on organic compounds for sustenance, these compounds support their population in the soil and remain stable over time. 

#### 11.6.4. Integrated Disease Management

**Figure 7:**
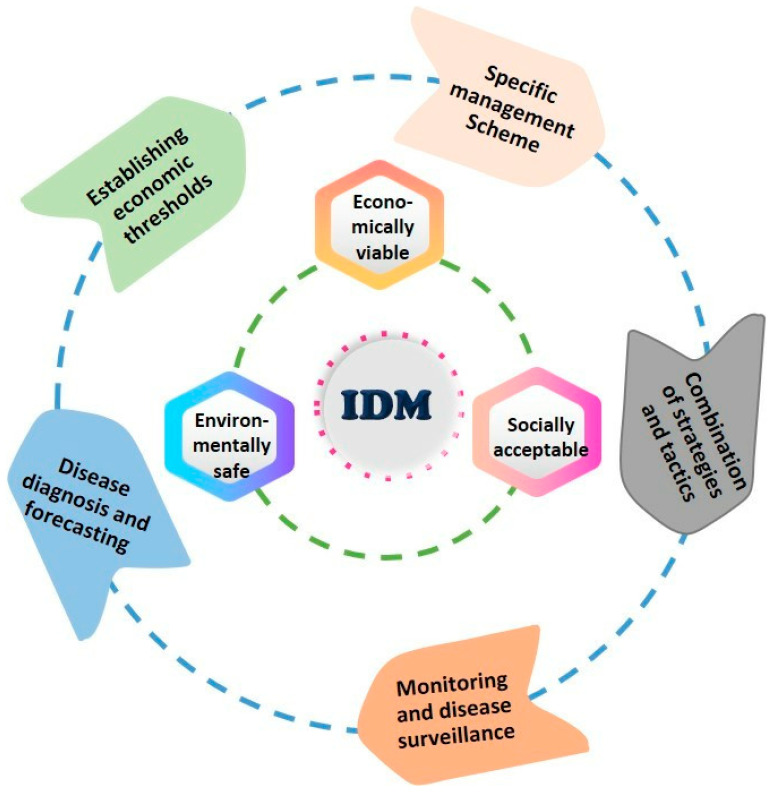
Integrated disease management (IDM) scheme. IDM involves a combination of management strategies that are economically viable, environmentally safe, and socially acceptable. IDM requires preventative measures, including disease monitoring and surveillance of multiple factors, accurate diagnosis, evaluation of disease threshold level, and guidance decisions on specific control measures and combining diverse strategies and tactics for sustainable disease management.

Although white mold can be controlled by various cultural, biological, physical, and chemical techniques, their levels of protection often fluctuate depending on the timing and conditions of their application. Eradicating *Sclerotinia* with a single control measure is challenging, making an integrated disease management (IDM) strategy the preferred approach. IDM combines cultural practices, varietal selection, chemical controls, and biological management to achieve adequate disease suppression [17]. The core principle of IDM is to move away from reliance on a single technology and adopt a more ecological approach that emphasizes understanding pathogen population biology. It also focuses on integrating readily accessible control components to support resource-poor farmers. It emphasizes the integration of control components that are accessible even to resource-poor farmers. Instead of aiming to completely eliminate the pathogen population, IDM seeks to manage it at commercially viable and environmentally sustainable levels. This strategy involves timely applications of multiple measures, including understanding disease factors, conducting disease surveillance, accurate diagnosis, disease forecasting, setting economic thresholds, and designing tailored management solutions (Figure 7). 

The primary benefits of IDM include promoting sustainable disease management alternatives and reducing the environmental risks associated with conventional practices by adopting ecologically based control tactics. A critical aspect of IDM is the synergistic interaction between various control agents and their compatibility with synthetic fungicides, ensuring effective integration into current disease management practices. Unfortunately, there is limited research on IDM techniques for managing *Sclerotinia*. However, some promising strategies have emerged. For example, combining low doses of fungicides with Contans^®^ has been shown to reduce labor costs while improving production efficiency in beans. A combination of Contans^®^ and Sumisclex 50 WP successfully suppressed white mold disease, resulting in 100% plant survival [131]. These findings suggest that combining Contans^®^ with fungicides at low concentrations is a cost-effective and efficient strategy for managing *Sclerotinia* in bean production.

The effectiveness of compost, chemical inducers, and fungicides, either alone or in combination, in controlling tomato white mold was investigated in the greenhouse and field conditions [171]. All treatments drastically decreased tomato white mold incidence and severity compared to the untreated control. Integrating salicylic acid and FeSO_4_ with compost resulted in the maximum increase in root and shoot weights, while combining salicylic acid with compost was most effective in reducing disease incidence and severity in the greenhouse. Under field conditions, integrating fungicide Billis and compost showed the utmost efficacy in reducing disease incidence and severity [171]. The efficacy of sawdust burning, fungal and bacterial bio-control agents, stable bleaching powder, Rovral 50 WP, and a combination of various components were evaluated to manage white mold disease of garden peas, mustard, and bush bean [172]. Integrating sawdust burning and soil amendments with *Trichoderma* bio-fungicide, *Bacillus* bio-control agents, and Rovral 50 WP appeared as the superior treatment, reducing 97.49%, 77.72%, and 72.26% white mold disease incidence and 84.61%, 81.14%, and 71.01% white mold disease severity in mustard, bush bean, and garden peas, respectively [172]. During Rabi 2018–2019 and 2019–2020, a field experiment was conducted to manage *Sclerotinia* stem rot of Indian mustard using an integrated disease management method [173]. As observed in the field, integrating seed treatment with Carbendazim (0.2%) and foliar spray of Carbendazim (0.2%) was the most effective, with an 81.82% reduction in white mold disease over the control, followed by combined application of seed treatment with Carbendazim (0.2%) and foliar Spray of Difenoconazole @ EC (0.1%)] with a 78.84% reduction in *Sclerotinia*. The yield appeared to increase by 58.06% and 52.24%, respectively, compared to the control [173]. Based on the findings, it is possible to conclude that integrating bio-control agents as a soil treatment and foliar application chemical fungicide is advantageous for controlling white mold disease caused by *S. sclerotiorum* and obtaining a higher yield under field conditions.

## 12. Conclusions and Future Prospects

The emergence of white mold disease and its widespread impact on crops across diverse broad-acre and horticultural farming sectors underscores the urgent need for innovative and effective management strategies. These strategies could include the registration and development of new fungicides, breeding disease-resistant cultivars, identifying reliable biological control agents, and leveraging biotechnological tools. While the development of fungicides with prolonged protective activity is time-intensive, it is critical for minimizing yield losses and preventing fungicide resistance. Though numerous in vitro studies have demonstrated the efficacy of various fungi, bacteria, and mycoviruses against *Sclerotinia*, success must be proven in planta and, more importantly, in field conditions where environmental factors significantly affect their performance. To enhance effectiveness, it is crucial to screen for more potent biocontrol strains with economic potential and to understand the mechanisms by which they operate. This knowledge can lead to better formulation and delivery strategies. Despite extensive research on QTL mapping for white mold resistance in different hosts, such resistance is rarely incorporated into breeding programs. This is due to the considerable time and effort required to develop disease-resistant cultivars and the limited expression of resistance under actual field conditions. In these cases, disease screening methods must align with the real-world growing conditions in the field. Furthermore, assessments of disease resistance should occur during the growth phase when the disease is most likely to manifest. The potential for genetically engineered crops to achieve complete resistance to *Sclerotinia* is an exciting prospect. Recent advancements in molecular biology and gene-editing technologies, such as CRISPR/Cas, offer promising opportunities to enhance host resistance to white mold. Finally, integrating these disease control measures with cultural practices could provide a multifaceted approach, combining potentially additive or synergistic modes of action against the pathogen, leading to a more robust and comprehensive defense.

## Figures and Tables

**Table 1:** Clinical symptoms and signs of white mold disease on various host plants.

Host Plant	Symptoms	Signs
General (All Plants)	− Dark brown, water-soaked patches on infected plant parts − Soft rot leading to tissue death − Wilting of terminal stems − Lodging of infected stems (in later stages)	− White cottony mycelium on plant parts and soil surface − Sclerotia visible on infected plant surfaces and inside dead tissues
Sunflowers	− Rapid wilting before/during blossoming − Stem rot at any growth stage − Rotting and crumbling of the inner sunflower head	− Formation of sclerotia in the infected stem and the head
Canola	− Early flowering followed by wilting of terminal stems − Blanched, darkish lesions on stems, branches, or pods − Lodging during seed filling	− Large black sclerotia inside the cortex of infected stems
Salvia	− Symptoms visible during blossoming − Symptoms begin on flower petals and spread to the entire bloom and lower flower parts	− Large dark sclerotia visible on and inside the infected plant tissues
Marigolds	− Symptoms start during blossoming − Spread from flowers to stems and foliage − Bleaching and collapse of infected stems	− Sclerotia observed within dead tissues
Cosmos	− Wilt, stem rot, and eventual plant death	− Sclerotia observed within dead tissues

Notes: Apothecia formation occurs on the soil surface under cool and wet conditions, often during the foggy winter season.

**Table 3:** Showcasing major fungicide groups effective against *Sclerotinia sclerotiorum* with the mode of action.

Fungicide Group	Important Fungicide	Types of Fungicides	Mode of Action
Anilinopyrimidines	Cyprodinil, mepanipyrim, and pyrimethanil	Contact	Inhibit methionine biosynthesis
Methyl benzimidazole carbamates (MBCs)	Benomyl, carbendazim, thiophanate-methyl, thiabendazole, and fuberidazole	Systemic	Inhibit cell division by disrupting microtubule formation
Dicarboxamides	Vinclozolin, iprodione, and procymidone	Contact	Inhibit osmotic signal transduction
Demethylation inhibitors (DMIs)	Imidazole, and propiconazole	Systemic	Inhibit membrane sterol biosynthesis and the development of functional cell walls)
Quinone outside inhibitors (QoIs) or strobil	Azoxystrobin, kresoxim-methyl, picoxystrobin, pyraclostrobin, and trifloxystrobin	Systemic	Block the transfer of electrons at the Quinone “outside” site of the bc1 complex (complex III in the electron transport chain)

## Data Availability

Not applicable.
